# True inclusion: evaluation protocol for an approach to increase meaningful participation of children with disability in athletics

**DOI:** 10.3389/fpubh.2024.1403866

**Published:** 2024-05-22

**Authors:** Abby Haynes, Simone Pearce, Kate Kirby, Dominique Moritz, Kerry West, Suzy Green, Christine McCoombes, Catherine Sherrington

**Affiliations:** ^1^Sydney Musculoskeletal Health, Institute for Musculoskeletal Health, The University of Sydney and Sydney Local Health District, Sydney, NSW, Australia; ^2^School of Law and Society, Sunshine Coast Mail Centre, University of the Sunshine Coast, Sippy Downs, QLD, Australia; ^3^Physiotherapy Department, The Children’s Hospital at Westmead, Sydney Children’s Hospitals Network, Sydney, NSW, Australia; ^4^Little Athletics Australia, Inclusion project, Albert Park, VIC, Australia

**Keywords:** children with disability, athletics, inclusion, contest, meaningful competition, fairness

## Abstract

Children with disability face many barriers to participating in community sports. Little Athletics Australia aims to increase fair and meaningful inclusion via a new structure which will enable all children to take part in the same contest by competing for their ‘personal best’ score. Named the True Inclusion Method (TIM), this new structure will be piloted in 13 sites across six states. Formative evaluation of this pilot will critique TIM and its implementation using observations of events, and interviews and surveys with child athletes with and without disability, their parents/carers and Little Athletics volunteers. Implementation outcomes are acceptability, appropriateness, adoption, feasibility and fidelity. Qualitative data will be analysed thematically. TIM is designed to encourage inclusive participation by children with disability in sporting events, and to improve the competitive experience for *all* children by celebrating personal achievement and fostering fun.

## Introduction

Sporting events provide valuable opportunities for children to have fun, engage in physical activity and forge connections in their community (1). However, children with disability face multiple personal, interpersonal and structural barriers to participating in sports, particularly competitive events, and are less likely to participate than children without disability (2–4). Diversity in children’s sporting competitions has widespread community benefits (5) and, for children with a disability, sport can play a valuable role in promoting dignity, equality and belonging (6). This is especially important given that many children with disability report feeling lonely and excluded, with limited social opportunities (7). Yet whilst most sporting organisations acknowledge that inclusivity is desirable, they seldom adjust the rules or structure of sport for children with disability and thus may fail to successfully foster *true* inclusion, i.e., equitable opportunities for children with disability to participate competitively in mainstream sport. Studies suggest that leaders in some sports clubs espouse the value of diversity but struggle to address it at a practical level, believing that inclusion of children and young people with disability is too hard and not core business (8, 9). Efforts to include children with disability may also be tokenistic (10), for example ‘just for fun’ or ‘have a go’ events where there is no opportunity to take part in meaningful competition. It is likely that established habits, systems and competing priorities, together with a lack of information and resources, compound the challenge of true inclusivity. This places the onus on children with disability and their families to ‘climb the fence to get in’ (11). Greater efforts are required to genuinely include children with disabilities in community sports. However, the concept of inclusion is contested and it is unclear what model might best support children of different ages and abilities to engage, and sustain enjoyment in, sporting competitions (12, 13).

This paper describes the protocol for a formative evaluation of a new model which aims to create conditions for *truly inclusive* participation by children with disability in a sports programme: Little Athletics, a form of athletics unique to the organisation Little Athletics Australia. It outlines an evaluation plan that aims to contribute to greater understanding of how children with disability can be welcomed into the athletics community (3).

### Little Athletics Australia

Little Athletics Australia (LAA) is the peak body responsible for governing a popular version of children’s athletics in Australia. It is a nationwide affiliation of state and territory associations that aim to encourage the development of children of all abilities by promoting positive attitudes and a healthy lifestyle through family and community involvement in athletic activities. To this end, LAA provides a sports programme for over 100,000 children aged 5–17 comprising modified track and field events tailored for those age groups delivered at approximately 500 local ‘Centres’ around Australia. Cross Country events are also sporadically offered by Centres depending on their location and accessibility to cross country courses. Competitive track and field events are held weekly for up to 26 weeks per year at ‘Centre meets’ where children generally compete in 4–6 events and are grouped according to age and gender. These groups may be combined physically when there are low numbers in a division, but the contest for each age and gender is recorded separately. Competition is structured to provide progressive pathways from weekly Centre competitions conducted at the grassroots level of the sport where rules are applied with some flexibility, to carnival, and regional or state level competitions where the contest is formal and rules are stringently applied. Between ten and hundreds of children are registered at each Centre, influenced by its location, surrounding population levels and demographics, and the type of events offered. At the Centre level, parents administer, conduct and coach the events in a voluntary capacity. Core values espoused by the organisation are inclusion, fun, innovation, integrity and ‘be your best’ (14, 15).

### Inclusion of children with disability at LAA

LAA has a history of including children with disability (CwD) primarily via the Multiclass system. Based on the Paralympic model for track and field classification of athletes with disability, specified by World Para Athletics (16), this system groups people according to the functional impact of their impairment. In Multiclass events, an athlete’s performance is ranked by calculating the percentage they scored against the “baseline” (based on world and national records) for their classification. This allows people in different classifications to compete against each other (16). In LAA, the inclusion of CwD operates differently at different levels of competition. At higher level competitions CwD generally compete in separate Multiclass events, providing they have a formal classification. These contests may be against other CwD in their age grouping or against all CwD at the carnival, or they might ‘compete’ on their own. At the Centre level, separate Multiclass events are relatively rare due to low numbers of CwD and the specificity of their classification, yet there is no other mechanism for recognising that CwD may be competing at a disadvantage. Modifications that might increase inclusion may be applied to some events at a Centre level (e.g., running along next to the hurdles rather than over the hurdles), but are not permitted at higher level competition Volunteers at Centres have variable levels of knowledge and confidence to apply these modifications and in some cases, meaningful modifications are not possible.

As the governing body for Little Athletics in Australia, LAA controls the rules at National events. Whilst state and territory bodies are permitted to implement their own rules and practises, they tend to adopt the National standards. However, when it comes to including CwD in events, there is considerable variation between and within states and territories. The project described here was developed with LAA to provide both a new structure of inclusive competition and greater consistency in how CwD are included. The project was developed and delivered with funding from the Department of Social Services (formerly the National Disability Insurance Agency) and Sports Australia.

### Limitations of current (and alternative) methods of inclusion

The True Inclusion project considers the inclusion of CwD in the *contest* of each Little Athletics event as distinct from the physical activity involved in that event. For example, a child with disability might be ‘permitted’ to run in the 100 m with all others their age, but a traditional first-past-the-post contest is not fair to them if they cannot compete from the same base capacity. The rules of the sport are designed to create a fair contest based on children having the same year of birth and gender. However, the creation of the Multiclass system recognises that this form of mainstream race is not fair to a CwD. Unadjusted inclusion in sports is an ableist approach because it reflects societal attitudes and structures that assume able-bodied standards, and does not make equitable accommodations for impairments to functional capacity that might allow for a fair contest (17).

The Multiclass system which is used in Little Athletics events seeks to remedy this situation. However, the Multiclass system itself has several limitations. First, it only captures some disabilities and levels of impairment and does not account for combined disabilities and/or cumulative effects of impairments or impacts that may arise from unclassified disability (18), thus it can privilege some competitors at the expense of others (19). For example, if a child who is legally blind has one eye scored marginally higher than the scores specified for the Vision Impaired class, she will have no classification category to be included in but will still have to ride tandem in triathlon. Second, the assessment process classifies CwD according to what is ‘wrong’ with them, a medicalised deficit approach which can be experienced as demeaning (6). Third, classification is time consuming and may be financially onerous for some families. Fourth, applying the Multiclass system to athletics events is complex and not always readily understood by local administrators, officials, parents and medical professionals so it can be applied incorrectly or inconsistently. Fifth, the Multiclass system is designed for adults, whereas children’s development is often in flux so the functional impact of their disability can change rapidly or differ from the trajectory of change anticipated by the classification criteria. The shorter review dates for children’s classifications do not adequately address this problem as access to professional classifiers can be limited. Sixth, the Multiclass system promotes segregated events for CwD which means these children can be the sole contestant or may be asked to compete against varied ages and genders, unlike their mainstream counterparts (18). Some argue that segregated events for children based on impairments can be viewed as ‘lesser’, reinforcing negative mainstream attitudes toward disability (20) and feelings of marginalisation by participants (21). However, CwD are diverse and there are differing views about the value of separate events for CwD and children without disability. For example, some CwD experience a greater sense of self-efficacy, belonging and communal support in disability-specific sports (22, 23). Choice is a critical factor, thus people with disability should have access to sporting activities that are inclusive *and* activities that are disability-specific (24).

Other structural characteristics of the current approach in Little Athletics also have a potential bearing on experiences of inclusivity by CwD. These include: an emphasis on competition and where children are placed in each event, the use of ‘levels’ or standards by which children determine how well they are progressing compared to the average participant, ‘best performer’ awards in and across events, and the promotion of ‘records’ for events at Centre, regional, state and national levels. To date, these structures have generally not recognised or responded to the disadvantage faced by CwD (18).

The result is that CwD do not have the same opportunities for meaningful competition in Little Athletics as children without disability. They are provided with less incentive to improve their fitness and skills, and fewer opportunities for social interaction and fun. Consequently, the Little Athletics community does not always benefit from the opportunity for children without disability to mingle with and take part in events together with peers who have a disability: activities which may reduce perceptions of disability as ‘otherness’ and increase wider social understanding and inclusion (25).

One alternative is to modify the structure of contests so that CwD are supported to compete against their mainstream peers within events more equitably by ‘levelling the playing field’. Examples that could promote greater competitive fairness include providing a head start for children with a disability, lighter throwing implements or lower hurdles. However, not all events are amenable to such modifications. For example, long jump is difficult to modify because its purpose is to identify who can jump the furthest. A child with a functional impact that impairs their ability to jump freely will be disadvantaged in that contest. Parents, who run these events voluntarily, may not have the skills or commitment to modify each event appropriately, and may fear that they are interfering with how the event ‘should’ be run (3). Crucially, modifications for CwD can also draw greater attention to a child’s impairments and to their differential treatment which may deter less confident children from participating (21). A further alternative is for children with disabilities to join in unmodified mainstream events with an adjustment to the final score that fairly reflects their disability. No mechanism for this score adjustment has been identified for CwD who currently compete in mainstream events in Little Athletics. It seems that revisions to the structure of Little Athletics events are required if CwD are to have real opportunities for fair and meaningful inclusion.

### The true Inclusion project

The True Inclusion project is a partnership between LAA and legal experts in disability and children’s rights at the University of the Sunshine Coast, working in collaboration with public health and physical activity researchers at the University of Sydney. Several team members have lived experience with disability and participation in little athletics as competitors, parents of competitors and volunteers. Little Athletics Australian Project Managers and Inclusion Officers are members of the team.

The project is informed by the human rights model of disability which highlights how policy, rules and regulations can disadvantage people with disability, and asserts that a person with disability should be provided adjustments to ensure they are treated on an equal basis with others (26). The project aims to co-design, implement and establish a support infrastructure for a competitive system that reflects the ethos of ‘substantive equality’ resulting in CwD having an experience that is no less favourable than their mainstream peer group (13). The objective is to create the foundations for treatment on an equal basis as outlined in article 30.5 (a) and (d) of the *UN Convention on the Rights of Persons with Disabilities* which states that signatories should take measures *‘To encourage and promote the participation, to the fullest extent possible, of persons with disabilities in mainstream sporting activities at all levels”* and *“ensure that children with disabilities have equal access with other children to participation in play, recreation and leisure and sporting activities’* (27).

The project commenced with stakeholder consultation and a systems mapping exercise which identified seven intervention strategies for tackling the individual, interpersonal and community level/organisational influences that shape the experiences of a child with disability in Little Athletics (28). The co-design and implementation of these strategies are core tasks for the project. They are to:

1. Identify and implement adjustments to Little Athletics structures and/or rules to enable fair and meaningful participation of CwD in contests with and against children without disability, obviating the need for Multiclass assessment and separate events.
2. Appoint dedicated champions within Little Athletics organisations—Inclusion Officers—who will promote and support inclusion strategies with colleagues and with athletes and their families.
3. Advise LAA member associations on the legal and other implications of current structures and processes for including CwD in Little Athletics.
4. Build the capacity of the Little Athletics workforce to deliver truly inclusive opportunities for CwD via training and resources.
5. Increase awareness of all Little Athletics participants and their families about new opportunities for inclusive participation in Little Athletics and the rationale for these developments.
6. Provide specialist information for CwD and their families about new opportunities for inclusive participation in Little Athletics.
7. Develop best practise and accreditation standards for LAA for true inclusion of CwD.

### The True Inclusion Method (the intervention being evaluated)

The True Inclusion Method (TIM) was designed to enable all child athletes to take part in the same event whilst competing for performance against their ‘personal best’ (manuscript in preparation). Each athlete’s performance is calculated and ranked compared to their previous personal best score; the winner is the child who surpassed their previous score by the most, or is closest to it. TIM can be applied to all children, both with and without a disability, and it can be used in combined events (e.g., pentathlons) and relays where it identifies the best all round athletes. Importantly, it can be implemented in parallel with traditional ‘first past the post’ contests enabling a dual system that may be maintained or used transitionally whilst the community becomes accustomed to alternative sporting structures. Essentially, TIM is a *personal best contest* which we labelled using the acronym TIM to enhance communication and engagement within Little Athletics about the model’s value in addressing inclusion.

TIM and the implementation strategies used to deliver it are informed by the system mapping described above which identified four domains of influence on competitive sports participation by children with disabilities: individual capabilities and circumstances; interpersonal dynamics; community/organisational norms and structures; and macro structures such as national funding guidelines, government policy and legislation. The design and implementation of TIM aims to impact the first three of these domains with the possibility of longer-term impacts on macro structures using evidence from the longer-term evaluation of TIM. Further details of TIM can be found elsewhere (15). This protocol focuses on the evaluation of the pilot implementation of TIM (Stage 1) as described below.

### Implementation plan outline

Stage 1 (pilot) of our implementation plan to support the delivery of TIM focuses on engagement and education/awareness raising, plus the provision of locally-determined support for Centres trialling TIM. Much of this work leverages the roles and skills of new staff members who were appointed to act as dedicated champions for true inclusion at the national level.

Five main implementation strategies will be used:

1. Engaging with relevant LAA stakeholders at executive and managerial levels (state and national levels) via education workshops, webinars and an expanded website aimed at providing ongoing capacity to promote true inclusion. This will be backed by a detailed report describing the rationale and development process behind TIM from legal and social perspectives, including a comparative explanation of the options reviewed by the research team and the factors considered in developing TIM as the recommended model for Little Athletics.
2. Providing clear and persuasive information to children and families who attend Little Athletics about TIM: how it works, what it aims to do and the rationale behind it. This will include flyers, information packs on TIM and a brief informational video. The video will be developed to capture attention and explain that CwD often feel left out of mainstream contests and that TIM provides a way for everyone to compete together with the same opportunities. The video will give examples of TIM being applied.
3. Providing education and resources about TIM and its rationale to the paid and volunteer workforce in the 11 Little Athletics Centres taking part in Stage 1 implementation. This will include presentations on TIM with discussion at Centre meets, consultation about how best to deliver TIM (and utilise the True Inclusion project team to support this delivery) in each Centre taking account of variations in local infrastructure and other resources, and a dedicated website with a growing range of resources aimed at providing ongoing capacity. See the Little Athletics Inclusion Hub for more details: https://www.littleathletics.com.au/inclusion.
4. Working with stakeholders and Timing Solutions (a company that specialises in results management software and timing for athletics and other sports events) to integrate TIM within the current Little Athletics results recording and reporting system known as Results HQ.
5. On-the-day event support from the True Inclusion project team to support the delivery of TIM. This will include explaining how TIM operates and what it is intended to do, identifying the results and how they relate to children’s personal bests, and demonstrating the outcome of that contest as compared to traditional contests.

## Methods

### Evaluation design

We will undertake formative evaluation to collate feedback from diverse stakeholders and learnings of project staff about early experiences of implementation, providing information that will enable us to modify implementation strategies and, if required, TIM itself, so that it better meets the needs of all stakeholders (29). The evaluation will focus on two related areas: stakeholders’ experience and perceptions of TIM (intervention evaluation) and critiquing the implementation strategies designed to support TIM’s uptake and maximise its effectiveness (implementation evaluation). These are described below.

**Figure 1:**
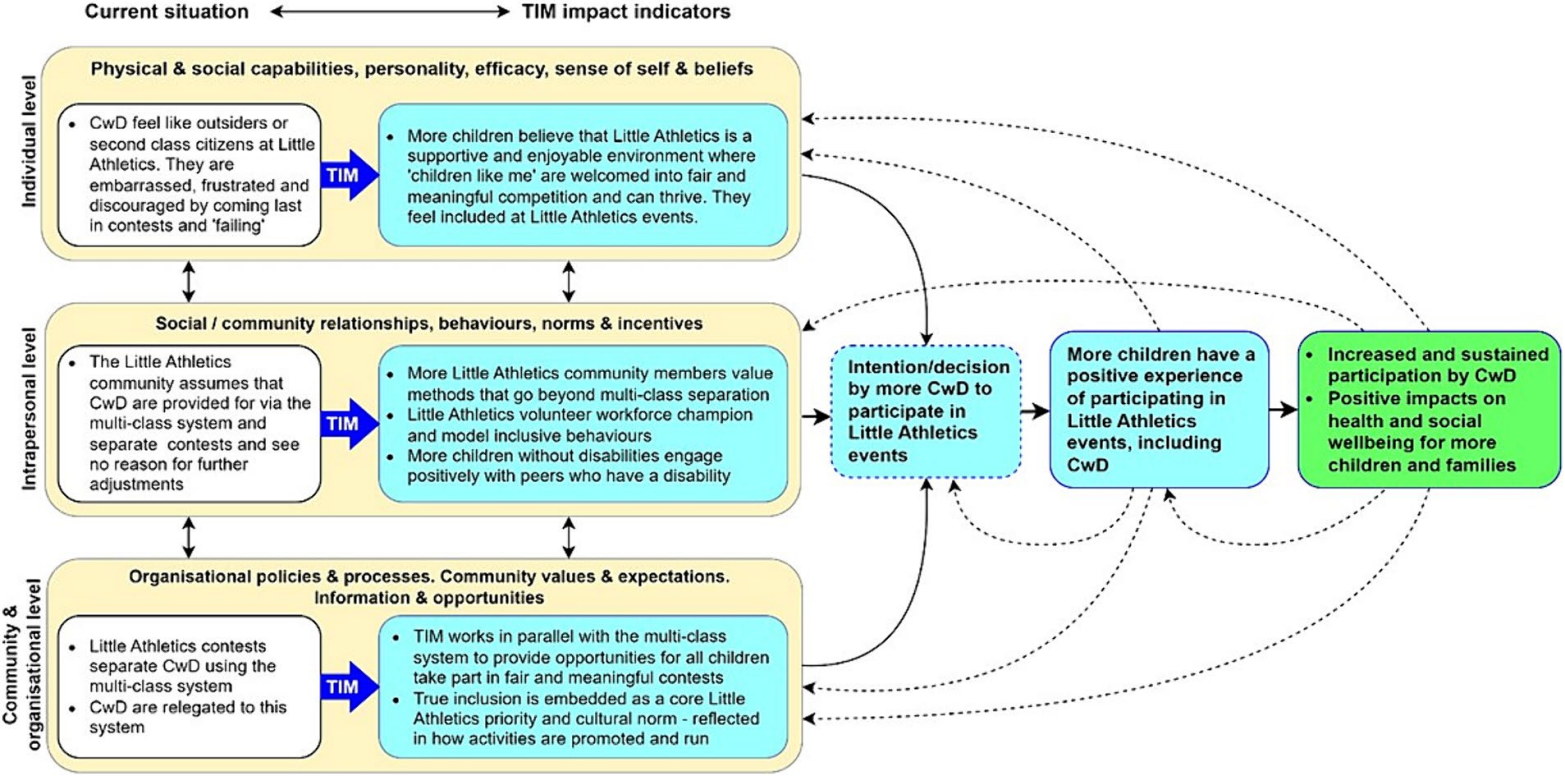
True Inclusion Method (TIM) program logic.

An initial programme logic model was developed to guide the identification of intervention outcomes and data collection targets for the evaluation (Figure 1) (30). This was informed by the systems mapping exercise described earlier and the theory of triadic influence [a meta-theory that consolidates multiple theories from social psychology, behavioural science and sociology (31)], and survey results from our own research which found that children with disabilities who participated in LAA events reported a lack of achievement, a sense of being “left behind” and difficulty participating in the full range of events offered.

This study is underpinned by a pragmatist research paradigm which is associated with producing actionable knowledge and promoting social justice (32). Pragmatic research recognises that meaning is dependent on human experience and it strives to understand this within historical, cultural and political contexts (33). Qualitative methods are most suitable for this form of inquiry (34). The design is informed by theory-driven evaluation (35) and implementation science methods (36). We will draw on ‘sensitising concepts’ that provide different ways of thinking about research questions and can guide how data is collected (e.g., by suggesting specific interview questions), analysed and interpreted (37). These concepts are critical disability theory with its emphasis on authentic inclusion and enabling/disabling social structures and environments (38, 39), and the role of “ableism” in disability discrimination in children’s sport (13, 40).

In keeping with the ethos of the True Inclusion project, the evaluation has been designed to align with Barnes’ (41) review of emancipatory disability research in that it aims to: (1) Include and be accountable to people with disability and their organisations, (2) Place the social model of disability at the heart of the research agenda, (3) Promote emancipation of people with disability and (4) Produce relevant research that has a meaningful practical outcome for people with disability.

### Settings

The 11 Little Athletics Centres taking part in Stage 1 implementation will be selected for maximum variation in: (a) local infrastructure to include Centres with electronic gates and manual recording, (b) socio-economic location/catchment, (c) regional/metropolitan location and (d) state or territory representation. This sampling strategy is intended to provide access to the widest range of experiences and contexts; however, some Centres have low numbers of participants so identifying sites where children with disabilities attend will be a consideration as their views and experiences are essential. Other considerations include the availability of suitable local events, research team resources and the potential impacts of COVID-19 on Little Athletics activities and travel options.

### Participants

Study participants will be members of the five key Little Athletics stakeholder groups:

1. Children with disabilities
2. Parents/carers of children with disabilities
3. Children without disability who attend Little Athletics
4. Parents of children without disability
5. The Little Athletics workforce: state based employees, Little Athletics Centre managers, committee members, Inclusion Officers, record keepers and volunteers.

There will be some overlaps between these groups; parents are often volunteers at Little Athletics and their family may include children with and without disability. See Table 1 for an overview of which groups will be targeted for different forms of data collection, and how they will be recruited.

**Table 1:** Overview of Stage 1 data collection plan: intervention evaluation.

Data collection method and participants	Data collection details including recruitment	Focus of data collection (including evaluation question/s targeted)	Time and place
Observation	Research team members, as observers, will move around each event venue informally seeking the widest range of observable information. Centre members will be sent information in advance about the process and consent obtained at the Centre level. No formal sampling of event attendees.	Consideration of how participation works, enabling/disabling systems and structures, TIM workability/feasibility in practise, and any relevant stakeholder dynamics and contextual factors, such as reactions to, or outcomes of, using TIM. Addresses evaluation question: 1, 3.	During events using TIM. In-person.
Anonymous online survey of parents/carers and children aged 10+	Research team members will hand out flyers about the survey to parents/carers and children aged 10 and over. The flyer includes a QR code that connects to the online survey. The survey will be brief (less than 5 min to complete) and mobile-friendly to enable on-site completion. Print copies of the survey will also be available.	Impressions of TIM and its comparison to the traditional LAA model, impact (including perceptions of fairness), contextual factors and any areas for improvement. Addresses evaluation questions: 1, 2, 4.	During or immediately after events using TIM. Recruitment in-person. Survey online.
Anonymous online survey of LAA volunteers	Research team members will hand out flyers about the survey to LAA volunteers. The flyer includes a QR code that connects to the online survey. The survey will be brief (less than 5 min to complete) and mobile-friendly to enable on-site completion. Print copies of the survey will also be available.	The attitudes and views of LAA volunteers regarding the workability, benefits and challenges of TIM, and their observations of how TIM includes children with disability in Centre meets. Addresses evaluation questions: 1, 2, 3, 4.	During or immediately after events using TIM. Recruitment in-person. Survey online.
Interviews with families (parents/carers and children aged 10+)	Research team members who hand out survey flyers will ask parents/carers if they are interested in taking part in a family interview at a time and location that suits them (via Zoom if required). Seeking approximately 12 family interviews overall. If possible, half would include families of children with disability.	In-depth exploration of experiences of participating in an event using TIM and how it compares to the traditional LAA model, impact, contextual factors and any areas for improvement. Special attention will be paid to whether TIM impacts children’s perceptions of fairness and inclusivity, fun, stigma and keenness to take part in future TIM events. Addresses evaluation questions: 1, 2, 3, 4.	Soon after events using TIM. In-person.
Collation of routine LAA data	n/a	Demographics, attendance figures, and TIM ResultsHQ software data. Addresses evaluation question: 3.	Before and after TIM events. Online.

### Intervention evaluation: stakeholder experiences of TIM

This will focus on understanding stakeholder experiences and perceptions of TIM during Stage 1. Our intervention evaluation questions are:

1. What are stakeholders’ perceptions of TIM? Specifically, do they believe that it is:
  - Workable/feasible, including sufficiently easy to administer?
  - Effective in providing fair and meaningful athletics competition opportunities for CwD within mainstream events?
  - Effective in providing fair and meaningful athletics competition opportunities for *all* children?
  - Superior in comparison to current models in providing more inclusive ‘equal basis’ athletics opportunities (when used in parallel with current models)?
2. What impacts do stakeholders report based on their experiences of TIM? E.g. do CwD feel more welcomed or valued? Are they mixing more with children without disability? (How) do Little Athletics volunteers feel their roles are affected?
3. How are the above affected by contextual factors such as geographical location, Centre resources, competition level and the ways in which Little Athletics structures and processes may act as enabling or disabling environments?
4. What suggestions do stakeholders have for how TIM might be improved and/or better integrated into Little Athletics events?

### Implementation evaluation: stakeholder experiences of implementation strategies and capacity building designed to support uptake and delivery of TIM

This part of the evaluation aims to monitor, explore and critique Stage 1 implementation strategies and capacity building with a view to identifying what is working well and what improvements would be beneficial. It targets the Little Athletics workforce (managers and other employees, and volunteers across all roles) in the 11 targeted Stage 1 Centres, focussing on five implementation outcomes: acceptability, appropriateness, adoption, feasibility and fidelity. These are described in Table 2. The questions guiding the implementation evaluation are:

1. What is the Little Athletics workforce’s perception of the information and support provided to increase the uptake and effectiveness of TIM? Specifically, do they believe that it:
2. Has content relevant to the needs of the Little Athletics workforce and is sufficient in supporting capacity to deliver TIM?
3. Is persuasive in making the case that TIM is superior in comparison to current models in providing more inclusive ‘equal basis’ sporting opportunities (when used in parallel with current models)?
4. To what extent has TIM been adopted in the targeted sites following the Stage 1 roll out?
5. What suggestions do members of the Little Athletics workforce have for how education and capacity building might be improved, or for any other supports/resources that would help them deliver TIM and support true inclusion effectively?

**Table 2:** Overview of data collection plan: implementation evaluation.

Implementation outcomes*	Implementation outcome measures	Source and data collection methods
Acceptability	Stakeholder understanding of TIM and the rationale for its uptake, and satisfaction with and belief in TIM itself.	All stakeholders: surveys and interviews as per Table 1.
Appropriateness	Stakeholder belief that ‘true inclusion’ is right for LAA, and that TIM is a good approach for achieving this.	
Feasibility	The perceived workability of implementing the capacity building strategies and delivering TIM across all LAA Centres.	LAA volunteer workforce: survey as per Table 1 (and focus groups planned for Stage 2)
Adoption	TIM is used by every Stage 1 Centre in at least one contest within these four event categories: Track (brief), track (distance), throwing, jumping. TIM features in the LAA 2022 strategic plan and other relevant documents. All Centres display TIM results in ResultsHQ once results are recorded (acknowledging that some results will be displayed instantaneously whilst others will be delayed).	Review of ResultsHQ records for each Stage 1 Centre over two consecutive meets Observations onsite by researchers and LAA Inclusion Coordinators. Collation of feedback from Centres at follow up by the research team.
Fidelity	True Inclusion team members introduce TIM, hand out flyers, collect results (if required) and input results into ResultsHQ (if required). Printed results are displayed during the meet with True Inclusion team members available to explain TIM results to athletes and parents. Centres have results available on ResultsHQ for 1 week. Results show the athlete’s typical position, time/distance recorded, if it was a PB, TIM position and TIM difference (difference in PB).	Observations onsite by researchers and LAA Inclusion Coordinators Review of ResultsHQ records.

*Adapted from Proctor et al., (36). Outcomes for implementation research: conceptual distinctions, measurement challenges, and research agenda.

### Data collection

Table 1 provides an overview of the data collection methods proposed for the intervention evaluation, whilst Table 2 provides an overview of the data collection methods for the implementation evaluation. Observations, formal interviews and focus groups will be conducted by one or more researchers with qualitative experience. Times and venues for interviews will be negotiated with participants. Where possible, participants will have the option for face-to-face meetings, but it is anticipated that videoconferencing will be favoured in most cases for convenience and minimisation of COVID-19 risks. Interviews and focus groups will be audio recorded using a digital recorder or videoconferencing software. Onsite observations of events will be captured via fieldnotes. Audio files will be transcribed verbatim by a research assistant. The researcher who conducted the interviews/focus groups will correct all completed transcripts.

### Data analysis

Transcripts and any written field notes will be uploaded to an appropriate qualitative data software management tool such as NVivo (42) and tagged by event, participant type, etc. We will then use a reflexive thematic approach to analyse the data. Thematic analysis is a theoretically flexible interpretative approach that focuses on identifying patterns in qualitative data sets and developing themes that reflect underlying narratives (43). This approach recognises that researchers are active contributors in knowledge production (44).

Data will be coded both inductively (i.e., codes will be applied to capture concepts that participants introduced) and deductively (i.e., a coding framework which is informed by our logic model, research questions and sensitising concepts will be applied to the data so we can identify the range of views that relate to each of the concepts). Two people will be involved in early coding to enable reflexive discussion. They will present emergent findings to the wider multidisciplinary research team who will act as ‘critical friends’: challenging and refining these themes and providing alternative interpretations (45). Analysis will commence soon after data collection starts. Early findings may be used to revise the data collections instruments (e.g., by identifying a new theme that we wish to explore using more targeted questions) and/or sampling (e.g., we may identify the need to find alternative methods of reaching a key stakeholder group).

The final results will be presented in a narrative comprised of key themes illustrated by deidentified quotes from participants, organised to answer our research questions. No individual or Centre will be identifiable in our reported findings unless they have given express permission for this. All members of the research team will contribute to the final presentation of results in publications.

This project was granted ethical approval by University of the Sunshine Coast human research ethics committee, approval number: A201466. All participants will provide informed consent.

### Stage 2 of TIM implementation

The Stage 2 implementation plan is still in development and will be informed by a review of pilot data from Stage 1. This may result in the redesign of components for optimal delivery of TIM. We plan to invite up to 10 Little Athletics Centres to use TIM through four-to-six of their regular Centre meets with support from the True Inclusion research team in adapting the method to align with local infrastructure, resources and community profiles, including integration into Results HQ or other technology used by each Centre to deliver results. However, no on-site support will be offered before or during Centre meets. Experiences with TIM using this less ‘scaffolded’ approach to delivery will be evaluated through a survey of the five key LAA stakeholder groups previously described, with a view to long term sustainability.

### Dissemination plan

The results of this research will be reported in academic publications and/or conference presentations, including at the Australian Little Athletics Championships conference. We will prepare a plain language report for LAA stakeholders and plan to conduct workshops or other presentations for Centre volunteers to share and discuss learnings from this research. Results may be used in opinion pieces and advocacy. Learnings from stages 1 and 2 may also be used in a series of workshops/webinars designed to provide education about inclusion for paid staff and the broader LAA Community. An online *Inclusion Hub* will offer practical strategies, tools and information.

## Discussion

The True Inclusion project aims to change the structure of competitive sporting events within Little Athletics, addressing inadequacies of the Multi-class system by providing a fair and meaningful alternative to segregated events. To this end, TIM was co-designed to provide sporting opportunities that can be enjoyed by all children equally. This evaluation will show to what extent our five stakeholder groups find the system worthwhile and feasible as consumers and deliverers, including the extent to which it successfully contributes to truly inclusive sporting opportunities. It will identify areas for improvement in the system itself and in how it is delivered, and enable us to fine-tune the strategies we use to build capacity, resource and otherwise support the Little Athletics workforce to implement this initiative at scale.

It is envisaged that proposed changes to the structure and rules of Little Athletics to address the disadvantage of children with disability may invoke wider political and social debate about the impact of traditional structures and rules in competitive sport and their role in creating ‘disabling environments’ or failing to provide substantive equality. We hope to leverage this attention via opinion pieces and advocacy. Forums such as The Conversation, Sports Bulletin (Lexis Nexis), school sports department publications, and national conferences in sport and education provide platforms for robust discussion about the discriminatory nature of traditional sporting competition structures, and the merits of truly inclusive structures and of TIM as a preferred approach for enhancing the Little Athletics experience for CwD and, indeed, all children.

We believe TIM has the potential to improve the experience of sporting competition for a diverse range of children—both with and without a disability—because it addresses some key barriers to sports participation identified by children more broadly. Studies consistently show that the majority of children involved in sports drop out by the time they reach puberty due to reasons that include lack of fun, loss of autonomy and perceptions of low physical competence (11, 46–48). Traditional first past the post contests may contribute to this dropout given that the pressure to succeed can foster a ‘win at all costs’ culture that overshadows other valuable purposes of sport such as making friends, developing a community identity, feeling strong and healthy, improving confidence, and developing personal attributes and abilities (11). Children themselves appear to share this view; for example, a US study found that over 90% of children who participated in sports did so because it was fun and they ranked winning as only the 48th most important reason for participation (46). Spaaij et al. (49) found that a focus on competitiveness at junior sports clubs’ negatively affected commitment to diversity and inclusive participation, suggesting that an orientation to pleasure/participation rather than power/performance contributes to a culture of greater inclusivity. It follows that where sport is focused on competition and does not offer fair and meaningful prospects to participate in that competition, many children will not want to take part.

TIM aligns with recommendations for reversing an emphasis on winning as the central focus of competitive sports for children (50). It redefines sporting goals, celebrating personal achievement (i.e., self-improvement) and fostering fun rather than the pressure to win. The focus on each individual’s personal goals gives children greater ownership of their competitive experiences, and it opens up the playing field with rules that give every child an opportunity to participate meaningfully. Elliot argues that the COVID-19 pandemic has created a window of opportunity to ‘reimagine’ children’s sport so that it is more fit-for-purpose in addressing mental wellbeing and physical activity, increasing family and social connectedness, and attracting new volunteers and participants into sport, instilling them with values and philosophies that can sustain lifelong engagement in sports (51). TIM may be one of these opportunities.

## Conclusion

This protocol describes the True Inclusion Method (TIM), its implementation within Little Athletics Australia and plans for a formative evaluation to critique and strengthen the method. We hope this work will contribute to changes in sporting events that recognise the disadvantages faced by children with a disability. Our model seeks to provide fair and meaningful opportunities for participation that increase engagement and fun, reduce stigma and stereotyping in the sporting community, and signal to children with disabilities that they are worthy of true inclusion.

## Data availability statement

The original contributions presented in the study are included in the article/supplementary material, further inquiries can be directed to the corresponding author.

## Ethics statement

This project was granted ethical approval by University of the Sunshine Coast human research ethics committee, approval number: A201466. All participants will provide informed consent.

## Author contributions

AH: Writing – review & editing, Writing – original draft, Visualization, Methodology, Conceptualization. SP: Writing – review & editing, Supervision, Project administration, Funding acquisition, Conceptualization. KK: Writing – review & editing, Project administration, Methodology, Investigation, Formal analysis, Data curation. DM: Writing – review & editing, Funding acquisition, Conceptualization. KW: Writing – review & editing, Investigation. SG: Writing – review & editing, Resources, Project administration, Investigation. CM: Writing – review & editing, Resources, Project administration, Investigation. CS: Writing – review & editing, Methodology, Conceptualization.
